# Correction: Guo et al. A Mixture of T-Cell Epitope Peptides Derived from Human Respiratory Syncytial Virus F Protein Conferred Protection in DR1-TCR Tg Mice. *Vaccines* 2024, *12*, 77

**DOI:** 10.3390/vaccines13040394

**Published:** 2025-04-08

**Authors:** Hong Guo, Yang Song, Hai Li, Hongqiao Hu, Yuqing Shi, Jie Jiang, Jinyuan Guo, Lei Cao, Naiying Mao, Yan Zhang

**Affiliations:** 1 NHC Key Laboratory of Medical Virology and Viral Diseases, National Institute for Viral Disease Control and Prevention, Chinese Center for Disease Control and Prevention, Beijing 102206, China; gh199408@163.com (H.G.); candyalbarn57@126.com (Y.S.); wslihai@126.com (H.L.); huhq049@163.com (H.H.); shiyuqing1997@yeah.net (Y.S.); jiejiang0317@163.com (J.J.); guojy@ivdc.chinacdc.cn (J.G.); caolei@ivdc.chinacdc.cn (L.C.); 2 National Key Laboratory of Intelligent Tracking and Forecasting for Infectious Diseases (NITFID), National Institute for Viral Disease Control and Prevention, Chinese Center for Disease Control and Prevention, Beijing 102206, China

The authors would like to make the following corrections to this published paper [1].

In the original publication, due to oversight, there was a mistake in Figure 2F. Figure 2F was intended to demonstrate the IFN-γ/IL-4 spot-forming units (SFUs), but the results incorrectly show the SFUs of IL-5. The corrected Figure 2 appears below. We also discovered a miscalculation in section “4. Discussion”, second paragraph, line 7, where the ratio between different groups had been calculated incorrectly. The sentence should be corrected to “In particular, the secretion of IFN-γ in the DR1-F9 group was 17 times higher than in the DR1-ADJU group, and the ratio of IFN-γ/IL-4 was 6.4 times higher than in the DR1-ADJU group”.

The other elements of the figure remain the same, and the interpretation of the results remains unchanged.

The authors state that the scientific conclusions are unaffected. This correction was approved by the Academic Editor. The original publication has also been updated.

## Figures and Tables

**Figure 2:**
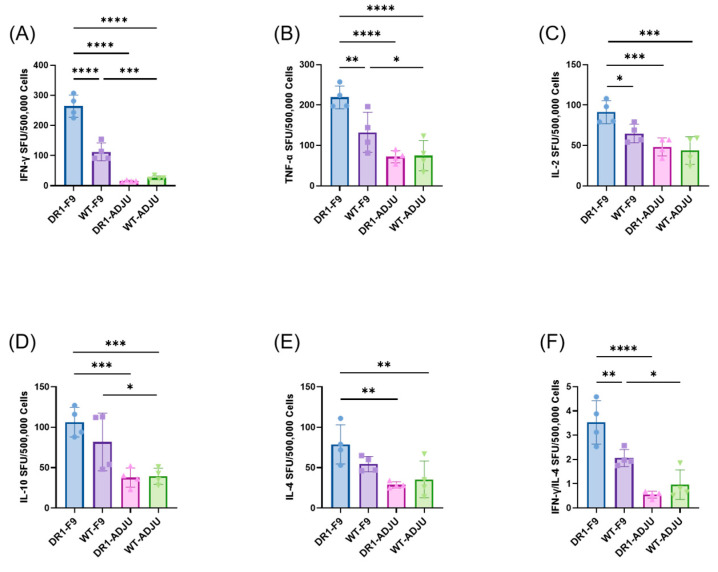
Mice exhibited Th1-biased T-cell responses. Mice splenocytes harvested 4 days after HRSV challenge were stimulated with F-9 PV for (**A**) IFN-γ spot-forming unit (SFU), (**B**) TNF-α SFU, (**C**) IL-2 SFU, (**D**) IL-10 SFU, (**E**) IL-4 SFU, and (**F**) IFN-γ/IL-4 SFU, respectively. Points represent individual mice. Statistically significant differences were measured by one-way ANOVA with Fisher’s LSD test. **** *p* < 0.0001, *** *p* < 0.001, ** *p* < 0.01, * *p* < 0.05.
