# Supplementary figures and images for: A Mixture of T-Cell Epitope Peptides Derived from Human Respiratory Syncytial Virus F Protein Conferred Protection in DR1-TCR Tg Mice
Source: Vaccines (Basel). 2024 Jan 11;12(1):77. doi: 10.3390/vaccines12010077 (PMC10820450; doi:10.3390/vaccines12010077)

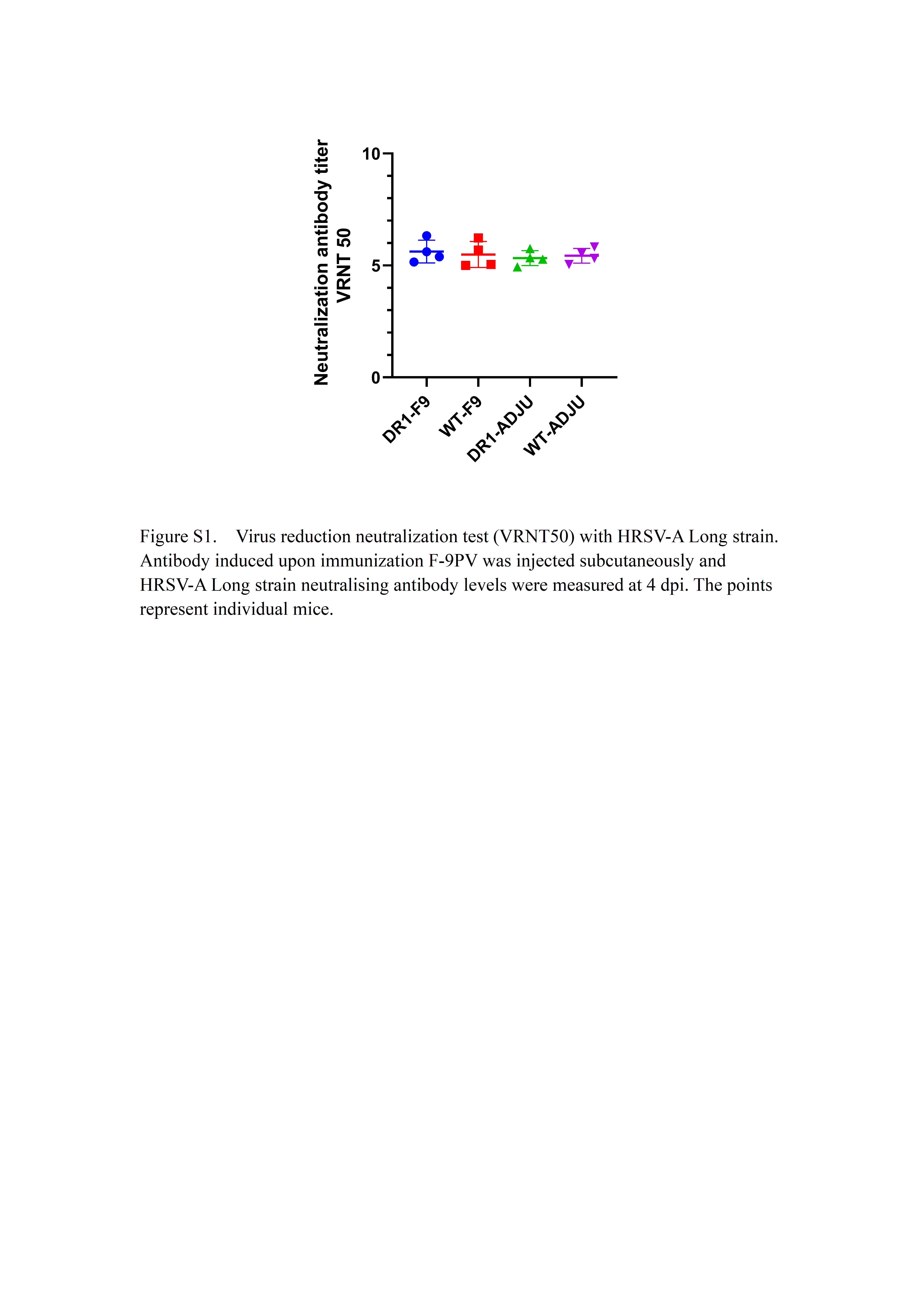

Supplement: Supplementary file 1 [file vaccines-12-00077-s001.zip › Supplementary files/figure S1.png]
